# Tubular Adenoma Arising Within a Rectosigmoid Xanthoma: A Case Report

**DOI:** 10.7759/cureus.62224

**Published:** 2024-06-12

**Authors:** Kaley Coffey, Heather L Mateja, William Bowers, Peter DeVito

**Affiliations:** 1 General Surgery, Trumbull Regional Medical Center, Warren, USA; 2 General Surgery, American University of Antigua, Osbourn, ATG

**Keywords:** lipid metabolism, colorectal polyp, tubular adenoma, colonoscopy, colonic xanthomas

## Abstract

Colonic xanthomas are a rare finding, particularly when combined with a tubular adenoma in a single polyp. While transformation to malignancy is not thought to be higher than that of a tubular adenoma alone, there is still concern as to the pathophysiology of xanthoma formation within the colon and what that may mean for patient outcomes. Here, we present a patient undergoing a routine screening colonoscopy with the removal of a rectosigmoid polyp consistent with xanthoma and tubular adenoma histopathology. Proper follow-up for identification of possible metabolic derangements and increased colonic surveillance is recommended to mitigate the risk of further xanthoma or adenocarcinoma formation.

## Introduction

A xanthoma is a proliferation of lipid-laden macrophages, which are most commonly found on the skin or deeper within soft tissues and often represent an underlying systemic disease [1]. However, they have been described within other organ systems and can occur independently from hyperlipidemias [1]. Gastric xanthomas have an incidence of 1-4% and are usually associated with gastritis with or without concomitant *Helicobacter pylori* infection [2,3]. Even more rare, with an unknown incidence, are colonic xanthomas, which are most frequently present in the rectosigmoid area [4]. It is unclear why xanthomas form within the colon of normocholesterolemic patients, although one theory suggests aberrant cellular regeneration after damage to the colonic mucosa, similar to the proposed mechanism for gastric xanthomas [1,4]. Here, we present a case of a rectosigmoid tubular adenoma containing foamy histiocytes consistent with a xanthoma on routine colonoscopy.

## Case presentation

A 58-year-old obese female with past medical history of hypertension, gastroesophageal reflux disease, and past surgical history of loop electrosurgical excision procedure (LEEP), hysterectomy, and hip replacement presented for a routine screening colonoscopy. The patient has had two prior colonoscopies in 2013 and 2019 without significant findings. Her mother was diagnosed with colon cancer previously, though she could not remember what age she was diagnosed. She is a non-smoker and does not drink alcohol or use recreational drugs. Her last known lipid panel revealed mild elevations in cholesterol being managed with dietary changes: total cholesterol 211 mg/dL, low-density lipoprotein (LDL) cholesterol 137 mg/dL, high-density lipoprotein (HDL) cholesterol 51 mg/dL, and triglycerides 117 mg/dL.

**Figure 1:**
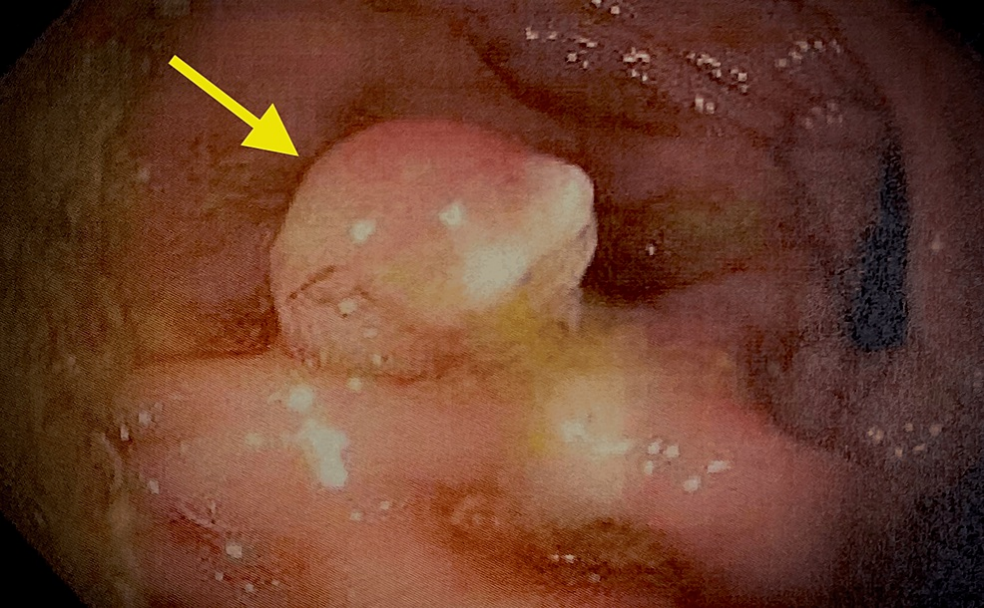
Colonoscopy finding of pedunculated colonic polyp specimen in the rectosigmoid area measuring 1 cm

A colonoscopy was performed without complications. The Boston Bowel Preparation Scale (BBPS) was a seven out of nine (two right colon/three transverse colon/two left colon), with a minimum of zero for each segment representing poor visibility and a maximum of three representing full visualization of the mucosa. The patient was noted to have extensive left-sided diverticulosis without complicated features. A 1-cm pink-tan rectosigmoid polyp was encountered at 20 cm (Figure 1).

A polypectomy was performed using loop snare cauterization, and histopathology of the mass reported a tubular adenoma with aggregates of lamina propria foamy histiocytes consistent with a xanthoma. The patient returned to the office for follow-up and was counseled on weight loss, with a recommendation to follow up with her primary care physician and repeat the colonoscopy in three years.

## Discussion

Histologically, xanthoma histiocytes are foamy macrophages containing intracellular lipid droplets, typically observed within the lamina propria, that stain positive with CD68 [4,5]. In contrast, tubular adenomas are characterized by dysplastic epithelial cells arranged in tubular structures with varying degrees of architectural complexity [6]. The coexistence of tubular adenoma and xanthoma within the same polyp raises questions regarding their pathogenesis and potential implications for disease progression. Few reports describe xanthomas and tubular adenomas arising from the same polyp, as in our case, and there is no clear reason to suggest transformation to carcinoma would be any more likely than in that of a singular tubular adenoma [4].

Colonic xanthomas are proposed to arise from regeneration of colonic tissue after cell damage generated by toxicity, as demonstrated by frequent mucosal involvement [3,7]. It is also theorized that there may be malignant transformation when the diacylglycerol from the foam cells is absorbed by the tissue and activates protein kinase C, an important player in signal transduction and growth regulation [4]. However, the more likely explanation for the formation and possible outcomes relate back to metabolic derangements of lipid metabolism. For example, cases of gastric xanthoma have been shown to disappear after treatment for hyperlipidemia [3]. Thus, a careful review of the patient’s overall health and proper surveillance of the colon should be considered upon detection of a xanthoma, particularly when associated with tubular adenomas or adenocarcinomas.

## Conclusions

Rectosigmoid polyps with xanthoma histiocytes represent a unique entity that poses diagnostic and management challenges for clinicians. Further research is needed to elucidate the underlying pathogenesis of these lesions and determine their clinical significance in the context of colorectal neoplasia. The management of rectosigmoid polyps containing xanthoma histiocytes depends on various factors, including the size, histological subtype, and presence of dysplasia within the adenomatous component. In cases where the polyp is small and low grade, endoscopic resection may suffice, with close surveillance recommended to monitor for recurrence. However, larger or high-grade lesions may warrant more aggressive intervention, such as surgical resection, to mitigate the risk of malignant transformation.
